# Surgical treatment outcome after serial debridement of infected nonunion—A retrospective cohort study

**DOI:** 10.1007/s00590-021-02930-4

**Published:** 2021-03-27

**Authors:** Markus Rupp, Stefanie Kern, Nike Walter, Lydia Anastasopoulou, Reinhard Schnettler, Christian Heiss, Volker Alt

**Affiliations:** 1 Department of Trauma Surgery, University Medical Center Regensburg, Franz-Josef-Strauss-Allee 11, 93053 Regensburg, Germany; 2 Department of Trauma, Hand and Reconstructive Surgery, University Hospital Giessen and Marburg, Campus Giessen, Rudolf-Buchheim-Strasse 7, 35385 Giessen, Germany; 3 Experimental Trauma Surgery, Justus-Liebig-University Giessen, Aulweg 128, 35392 Giessen, Germany; 4 Faculty of Medicine, Justus-Liebig-University Giessen, Klinikstrasse 23, 35392 Giessen, Germany

**Keywords:** Nonunion, Infection, Bone infection, Debridement, Surgery

## Abstract

**Purpose:**

Reported outcome after multiple staged surgical treatment of infected nonunion is scarce. We, therefore, asked: (1) What is the clinical outcome in infected nonunion patients after multiple staged revision surgery? (2) Are different pathogens evidenced after surgical treatment in patients who have undergone more or less surgeries?

**Methods:**

All enrolled patients were surgically treated for long bone-infected nonunion between January 2010 and March 2018. Besides patients´ demographics outcome in terms of bony consolidation and major complications defined as death during inward treatment, amputation and recurrence of infection during follow-up of at least 12 months were assessed. Microbiological findings were assessed and compared between two groups with less than five versus five or more surgical revisions.

**Results:**

Bone consolidation was achieved in 86% of the patients while complications such as femoral or transtibial amputation, recurrence of infection or even death during inpatient treatment could be evidenced in six patients (14%). In patients who underwent multiple-stage surgery for five or more times, germ changes and repeated germ detection was more common than in patients with less surgeries.

**Conclusions:**

Surgical treatment of infected nonunions poses a high burden on the patients with major complications occurring in about 14% of the patients using a multiple staged treatment concept. Future prospective studies comparing outcomes after limited with multiple staged revision surgeries are necessary.

## Introduction

Bone and joint infections are one of the most challenging complications in the orthopedic and traumatological field. With increasing incidences of arthroplasty procedures revisions for periprosthetic joint infection (PJI) will pose not only a significant burden to affected patients but also to the society due to its socioeconomic impact [1–3]. In addition, increase in numbers of often more complex fractures especially in older adults will result in an increase of fracture-related infections (FRI), posttraumatic osteomyelitis and nonunion [4].

The evolution of modern orthopedic and trauma surgery was coined by efforts to avoid and treat bone and joint infections [5]. In 1927, one year before Alexander Fleming discovered penicillin, the American surgeon H. Winnett Orr described a staged surgical treatment protocol for treatment of osteomyelitis. After exposure of the diseased bone, necrotic parts were to be removed, wounds were left open and gauze put into the wound. Dressings and plaster were changed as less as possible to avoid recurrence of infection [6]. Later in the twentieth century, this initial concept of staged surgical treatment was adapted to revision arthroplasty [7] and prevention of infection in open fractures [8]. Delayed wound closure was regarded necessary to allow successive wound debridement. Thus, avoidance of deep infections by clostridia species and other anerobic pathogens was deemed to be achieved best [9]. However, recent studies demonstrated promising results of reduced risk of deep infection and nonunion by thorough debridement and immediate wound closure after open fractures without routinely scheduled debridement and wound irrigation procedures [10–12]. Nevertheless, concepts of planned surgical interventions for bone debridement and wound irrigation in set time-frames are still recommended for surgical treatment of bone infections and widely accepted [13]. In chronic osteomyelitis, programmed wound irrigations continued until no pathogens are detected by microbiological analysis is still regarded being reasonable for surgical infect eradication [14]. Multiple staged surgical interventions for infect eradication in bone and joint infection are a very exhaustive psychological and physical burden for patients. Furthermore, costs due to frequent and long operations are high. We, therefore, asked: (1) What is the clinical outcome in infected nonunion patients after multiple staged revision surgery? (2) Are pathogens evidenced after surgical treatment different in patients who have undergone more or less surgeries?

## Methods

### Study design, patient enrollment and demographics

The ethics committee of our institution approved the study protocol, AZ 68/18. The study was planned as a retrospective cohort study. All patients enrolled to the study had to be operated for infected long bone nonunion between January 2010 and March 2018. Minimum age for inclusion to the study was 18 at time of revision surgery. Follow-up surgical debridements were planned based on surgical assessment of intraoperative bone and soft tissue status, which can be regarded similar to above-mentioned concepts of planned surgical revisions. We retrospectively reviewed patients´ medical records. Nonunion was defined by failure of fracture healing for at least six months. Patients with delay in fracture healing less than six months were excluded for the study. Also, patients who received an implant coated with antibiotics were excluded. Reoperation within six months after definitive fracture treatment was performed in 21 patients, which was not considered an exclusion criterion. Infected nonunion was diagnosed if one or more of the following criteria were present: the presence of a sinus tract, purulent discharge, exposed osteosynthesis material, positive “probe to implant” test, positive microbiological culture result, histologically confirmed infection (> 5 granulocytes per field of view at a magnification of 400), > 2000 leucocytes /µl in synovial fluid or > 70% granulocytes of cells in synovial fluid of concomitant septic arthritis according to the fracture-related infection consensus criteria [15]. Patients´ medical history and symptoms like erythema, swelling, rest pain and pain on weight bearing were considered as suggestive parameters for infected nonunion. Elevated infection parameters in laboratory tests (white blood cell count, C-reactive protein) and radiological signs of infection (osteolysis, implant loosening, sequester formation) were regarded as indicators for infected nonunion as well. However, diagnosis was not based on those findings. Following the 2018 international consensus meeting on musculoskeletal infection [15], FRI was confirmed by the presence of at least one of the following confirmatory criteria: (1) fistula, sinus tract or wound breakdown (2) purulent drainage or presence of pus during surgery, (3) phenotypically indistinguishable organisms identified by culture from at least two separate deep tissue/implant specimens (including sonication fluid) and (4) histopathological findings (presence of microorganisms in deep tissue specimens or presence of > 5 PMN/HPF in chronic/late-onset cases (e.g., fracture nonunion). Fractures were classified in accordance with the AO/OTA fracture classification [16]. Besides radiological fracture classification, fractures were classified as closed and open fractures. Microbiological culture results of intraoperatively taken tissue samples were analyzed. Tissue samples, sonication fluid of osteosynthesis implants or synovial fluid in case of joint involvement were used for cultures. At each revision, a minimum of 3 cultures was taken. Samples were cultured on solid agar plates such as Brain heart infusion (BHI) agar, Columbia agar and MacConkey agar plates at 37 °C. In general, a prolonged incubation time of fourteen days was performed to improve diagnostic yield. In case of negative culture results, additional reverse-transcriptase polymerase chain reaction (PCR) was carried out. Sonication of the implants was introduced in 2016 and performed as described before [17]. Initial proof of mono- and polymicrobial infections was determined. Consecutively determined pathogens during surgical treatment were registered as well. Patients´ medical records were searched for patient-specific characteristics such as gender, age, body-mass-index (BMI), comorbidities, American Society of Anesthesiologists (ASA) classification, medication at time of revision surgery and allergies. Laboratory tests prior to the initial revision surgery were assessed. As revision surgeries, only operations with bony debridement at the former fracture site, performed after six months of initial injury were considered. Smaller soft tissue interventions without bony debridement and thus deep tissue sampling for microbiological analysis were not assessed for further analysis.

Treatment characteristics were assessed such as (1) bony consolidation evidenced on conventional X-rays or computed tomography. (2) Number of all revision surgeries which included bony debridement at the former fracture site, (3) major complications defined as death, necessary amputation or recurrence of infection needing further surgical treatment.

### Statistical analysis

Data were analyzed using SPSS statistics version 24.0 (IBM, SPSS Inc., Armonk, NY). For analyses of differences between patients, the chi-squared *χ*^2^ test or Fiser’s exact test was applied for categorical variables. Wilcoxon’s signed rank test and the Mann–Whitney U-test were applied for between-group comparisons. A *p* value less than 0.05 was considered significant. Data were exhibited in graphs as means ± standard error of the mean (SEM).

## Results

### Patient demographics, number of revision surgeries, major complications

A total of 42 patients enrolled in this study were diagnosed with infected nonunion. Mean age of study cohort was 54 ± 18 years (range 23–95). Overall, 26 (62%) of the patients were male and 16 (38%) were female. Demographics of patients showed that infected nonunions occurred most often in patients with fractures at the tibia/fibula (62%, *n* = 26) compared with other fracture sites (humerus, radius/ulna, femur) (Table 1). Moreover, almost half of the patients with infected nonunion had a BMI > 30 kg/m^2^ (43%, *n* = 17) and 46% suffered an open fracture. Most patients with infected nonunions were classified as ASA class II (62%, *n* = 26) patients. Regarding patients’ demographics (Table 2), there was no statistical significantly association between gender and number of revision surgeries (*χ* ^2^(1) ≥ 0.01, *p* = 0.6). No correlation was found for localization of fracture (*χ* ^2^(3)  ≥  4.1, *p* = 0.3), ASA score (*χ* ^2^(2) ≥ 2.5, *p* = 0.4), BMI [kg/m^2^] (*χ* ^2^(2) ≥ 1.0, *p* = 0.6) and initial open or closed fractures (*χ* ^2^(1) ≥ 0.7, *p* = 0.5). Of the 42 infected nonunion patients, 36 (88%) had successful bone healing, while major complications such as femoral or transtibial amputation, recurrence of infection or even death during inpatient treatment occurred in 6 (14%) patients (Table 3). Mean number of revision surgeries required for infected nonunion was 7.7 ± 1.2.

**Table 1:** Demographic data and clinical characteristics of patients diagnosed with infected nonunion

		Infected nonunion [*n*/%]
Patients	Total number	42
Gender	Female	16/38
	Male	26/62
Fracture localization	Humerus	2/5
	Radius/ulna	3/7
	Femur	11/26
	Tibia/fibula	26/62
ASA score	1	2/5
	2	26/62
	3	12/29
BMI [kg/m^2^]	< 18.5	–
	18.5–25.0	14/35
	25.0–30.0	9/23
	> 30.0	17/43
Fracture	Closed	22/54
	Open	19/46
Postoperative outcome	Successful healing	36/86
	Major complications	6/14
Revision surgeries [*n*]	1–4	18/43
	> 5	24/57

**Table 2:** Comparison of demographic and clinical data of patients with 1–4 and > 5 revision surgeries

		Revision surgeries		Pearson *χ*^2^ test	
		1–4 [*n*/%]	≥ 5 [*n*/%]	[Value/df]	*p* value
Total		18/43	24/57		
Gender	Female	7/44	9/42	0.01/1	0.6
	Male	11/56	15/58		
Fracture localization	1 Humerus	2/11	–	4.1/3	0.3
	2 Radius/ulna	1/6	2/8		
	3 Femur	6/33	5/21		
	4 Tibia/fibula	9/50.0	17/71		
ASA	1	–	2/9	2.5/2	0.4
	2	13/77	13/57		
	3	4/23	8/35		
BMI [kg/m^2^]	< 18.5	–	–	1.0/2	0.6
	18.5–25.0	6/35	8/35		
	25.0–30.0	5/29	4/17		
	> 30.0	6/ 35	11/48		
Fracture	Closed	11/61	11/48	0.7/1	0.5
	Open	7/39	12/52

**Table 3:** Patient demographics and treatment characteristics of patients with major complications after revision for infected nonunions

	1	2	3	4	5	6
Complication	Relapse of infection after 2 years	Above knee amputation	Transtibial amputation	Reinfection after arthrodesis	Above knee amputation	Death, heart failure
Age [years]	85	57	34	58	40	69
Gender	Female	Male	Female	Female	Male	Female
ASA	2	3	3	2	2	3
BMI [kg/m^2^]	38.3	41.7	46.9	46.5	26.3	22.2
AO fracture localization	44-C1	42-C3	43-C3	44-C1	42-C2	41-A2
Fracture type	Closed	Open	Open	Closed	Closed	Open
Revision surgery [*n*]	6	5	18	7	2	5
Number of germs [*n*]	1	4	5	2	1	2
Microbial detection	Course of treatment	Course of treatment	Course of treatment	Course of treatment	First revision	Course of Treatment
Repeated germ detection	No	No	Yes	No	No	Yes
Germ-changes	Yes	Yes	Yes	No	No	Yes

### Microbiological findings

**Fig. 1:**
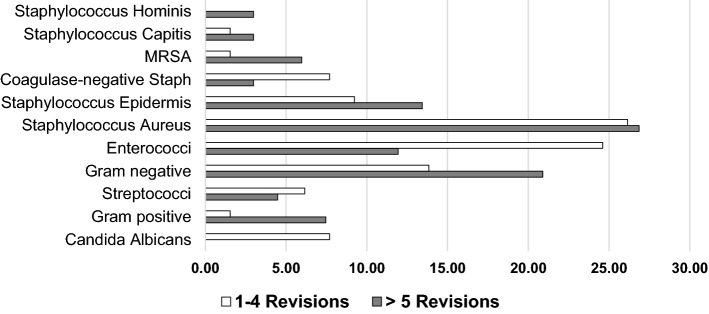
Differences in germ spectrum between patients with more and less than five revision surgeries. In both patient groups, *Staphylococcus aureus* was the most often identified pathogen. Gram-negative, other gram-positive, *Staphylococcus epidermis* and MRSA were more prominent in patients with five or more revision surgeries. In addition, *Enterococci* and *coagulase-negative staphylococci* were found more in patients with 1–4 surgeries

Overall, 125 pathogens were identified in all patients counting all surgeries. In patients with 1–4 (*n* = 18) revision surgeries 65 germs were detected in total. Main detected pathogen was *Staphylococcus aureus* (*n* = 17, 26%), followed by *Enterococci* strains (*n* = 16, 25%) and gram-negative bacteria (*n* = 9, 14%). 67 germs were detected in patients with more than five surgeries (*n* = 24). Here, *Staphylococcus aureus* (*n* = 18, 27%) was also the most identified germ, followed by gram-negative bacteria (*n* = 14, 21%) and *Staphylococcus epidermis* (*n* = 9, 13%). MRSA was detected more in patients with higher number of surgeries (*n* = 4, 6%) compared to patients with less than five surgeries (*n* = 1, 2%). Also, gram-positive bacteria and *Staphylococcus epidermis* were detected more often in patients with more than five surgeries (7% vs. 2%) (Fig. 1).

In addition, data analysis revealed major differences between infection characteristics of patients with 1–4 revision surgeries compared with patients with more than five surgeries (Table 4).

**Table 4:** Microbiological findings differed between both groups

		Revision surgeries		Pearson *χ*^2^ test	
		1–4 [*n*/%]	≥ 5 [*n*/%]	[Value/df]	*p* Value
Total		18/43	24/57		
Germ changes	No	17/94	4/17	25/1	*p* < 0.001
	Yes	1/6	20/83		
Microbial detection	Only first operation	17/94	1/4	34/1	*p* < 0.001
	Course of treatment	1/6	23/96		
Type of infection at first revision surgery	Monomicrobial	14/78	22/92	1.6/1	0.4
	Polymicrobial	4/22	2/8		
Repeated germ detection in course of follow-up surgeries	No	17/94	9/38	14/1	*p* < 0.001
	Yes	1/6	15/62		
Number of repeated germ detection in course of follow-up surgeries	1	–	–	3.5/3	0.4
	2	–	11/69		
	3	1/100.0	3/19		
	4	–	–		
	5	–	1/6		
	6	–	1/6		
Number of detected germs in course of surgical treatment	1	13/72	4/17	15/4	0.001
	2	4/22	8/33		
	3	1/6	8/33		
	4	–	3/13		
	5	–	1/4		

Only a few infected nonunions were due to polymicrobial infections. Both pathogen changes and repeated detection of the same germs could be evidenced significantly more often in patients who underwent 5 or more surgeries

**Fig. 2:**
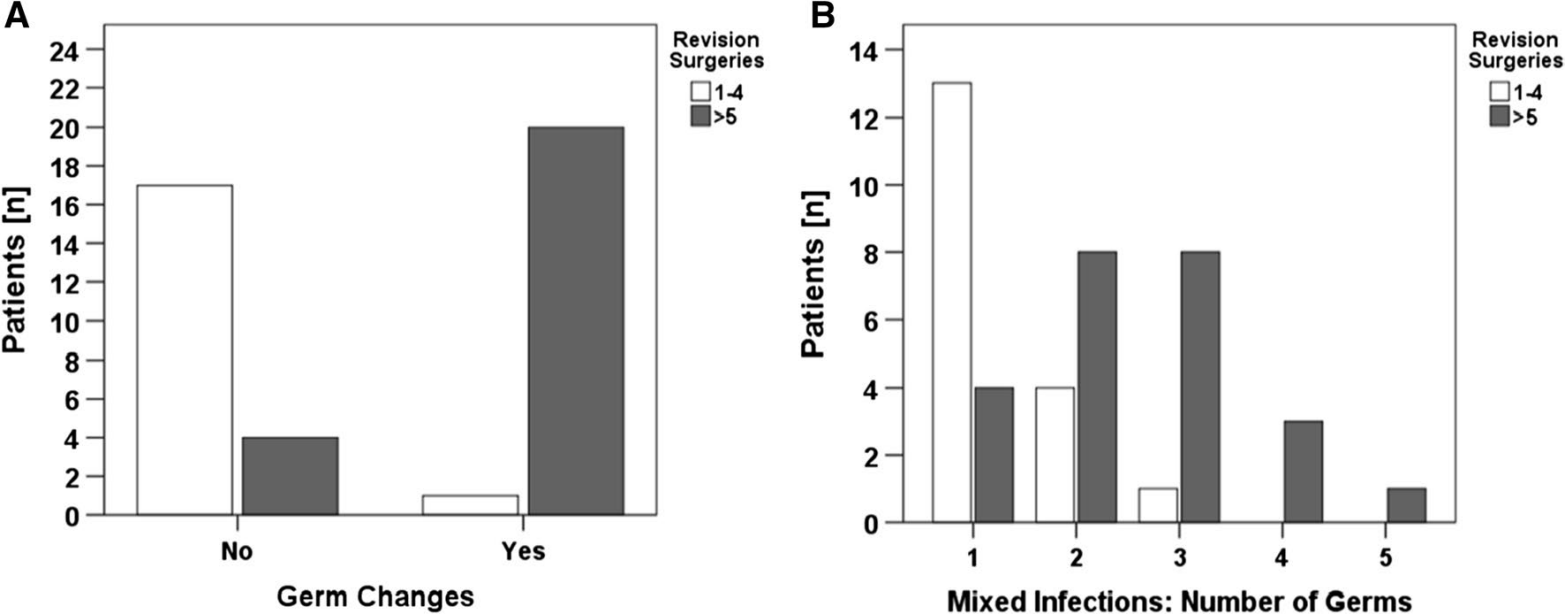
Germ changes (**a**) and number of different pathogens in course of the treatment (**b**) are associated with higher number of revision surgeries. **a** Germ changes appeared significantly more in patients with more than five revision surgeries *χ*^2^(1)  ≥  24.889, *p* < .001). Numbers of different pathogen correlated to higher number of surgeries (*χ*^2^(4) ≥ 14.991, *p* = 0.001)

In patients with more than five follow-up revision surgeries, pathogen changes were significantly more often detected (*n* = 20, 83%). In contrast, only one patient (*n* = 1, 6%) in the group of 1–4 revision surgeries had a change of evidenced pathogen during course of treatment. Thus, germ changes were associated with number of revision surgeries (*χ*^2^(1) ≥ 25, *p* < 0.001) (Fig. 2a). Looking at microbial detections, pathogens were detected in the patient group with 1–4 surgeries almost exclusively in the first revision surgery (*n* = 17, 94%). In patients with more than five surgeries, the contrary was seen. Microbial pathogens were detected also within the course of treatment in this group in 23 out of 24 patients (96%). Pearson’s *χ*^*2*^ test showed a positive association between time of microbial detection and number of revision surgeries (*χ*^2^(1) ≥ 34, *p* < 0.001). No association was found between number of revision surgeries and type of infection (mono- or polymicrobial) at the first revision surgeries (*χ*^2^(1) ≥ 1.6, *p* = 0.4). However, there was an association between number of surgeries and repeated detection of the same germ in follow-up surgeries (*χ*^2^(1) ≥ 14, *p* < 0.001). In addition, more revision surgeries led to several germ changes (*χ*^2^(1) ≥ 15, *p* = 0.001) (Fig. 2b).

## Discussion

In the retrospective cohort studied for surgical treatment strategy in infected nonunion, number of surgeries for bony debridement was considerable. Planned staged revision surgeries achieved good results in terms of limb reconstruction and bony union. In patients who underwent more sequential bone debridement the initially evidenced microbial pathogen was often found after following revision surgeries. In addition, germ changes could be detected significantly more often in the treatment group with more surgeries.

The burden of multiple staged surgery remains high. Time from first revision surgery to bony consolidation is long. This time of restricted weight bearing, wearing frames, multiple outpatient visitations as well as restrictions in social life are a high burden to bear for the affected patients. In the last two decades, surgical treatment concepts for FRI [18] and PJI [19] have evolved which both emphasize the significance of extended initial surgical debridement and supportive antibiotic treatment. Based on this evolution, even successful one-stage procedures for infected long bone nonunions [20, 21] and chronic osteomyelitis have been reported [22]. Wu and coworkers demonstrated successful two-stage management in Cierny-Mader type IV osteomyelitis. Only 5 of 36 patients required an unplanned second debridement in their case series. Intriguingly, all patients experienced bone defect healing. A high rate of eradication of osteomyelitis has been accomplished without major complications using a two-stage approach. Successful treatment with less but thorough debridement is in line with our findings [23]. Differences in patient characteristics in both small patient cohorts, anatomical localizations of the infected bone defects and procedures for bone defect reconstruction make comparing those outcomes with the present data difficult. Nevertheless, planned staged revision surgery in our historical cohort seems not to be superior to the two-stage approach described by Wu and coworkers. Furthermore, a limited surgery approach requiring less surgical interventions reduce not only the stress which infection patients are confronted during planned staged surgical treatment but also costs for the health care providers. Especially caregivers who underly lumpsum payment, which is customary in widespread remuneration systems based on the diagnosis-related groups (DRGs), reduced utilization of operation room capacities is of economic interest. Besides, less surgeries save personal and material resources in hospitals. In addition, avoidance of limb amputations is of socioeconomic interest. Successful limb reconstruction results in lower life time costs than amputation of the extremity [24]. Further, return to work is reported to be higher in reconstruction of the tibia compared to a group of amputees after high-energy open tibial fractures [25].

Repeated detection of the initially evidenced pathogens might be due to surgical circumstances. Staged revision programs with multiple surgical interventions might lead to less radical debridement which could be reason for pathogen detection during following surgeries. On the contrary, awareness that further revision surgeries which could be maleficent to the patient, could lead to a more thorough debridement of the operating surgeon. Changes in pathogens evidenced after consecutive surgical revision illustrate another therapeutic problem accompanying planned surgical revisions. Since PCR-based assays were used for microbiological examination, it is unlikely that pathogens detected in the course of the treatment were already present as viable but nonculturable pathogens during initial surgery. Although efforts to avoid surgical site infections have been made in the recent years [26], changes in evidenced pathogens after follow-up surgeries are most likely due to contamination during surgical revision. Since follow-up surgeries affect host´s innate and acquired immunity [27], surgical induced immunosuppression is likely to favor microbial superinfection due to repeated surgical interventions. For fracture healing, however, the clinical relevance of impaired host´s immunity due to numerous surgeries is not yet clear. Low numbers of initial polymicrobial infections in infected nonunions can be deemed favorable for infect eradication and nonunion treatment. Thus, changes in pathogens through additional planned surgeries might impede initial easier to treat microbial infections.

The major shortcoming of the study is its retrospective design. We focused on surgeries with bone debridement, to avoid a bias which might result from smaller soft tissue interventions and general different treatment approaches including all different options of bone reconstruction. During the observation period, which covers eight years, no fixed intervals for revision surgeries did exist. Surgical debridement was planned by senior consultants´ assessment of bone and soft tissue status. Including all different kinds of long bone-infected nonunions and different treatment options resulted in heterogeneity of the study group. To answer the question if particular frequent debridements influence germ patterns evidenced by microbiological examination, we decided to compare two similar-sized groups. To achieve this goal of similar-sized groups, patient cohorts with more or less than five bony debridements were set. This cut-off was deemed reasonable since revision surgeries included the final revision surgery after infect eradication.

## Conclusion

The examined topic of multiple staged surgical revision for infections is of the highest clinical relevance for all surgical entities. Surgical treatment of infected nonunions poses a high burden on the patients with major complications occurring in about 14% of the patients using a multiple staged treatment concept. Future prospective studies comparing outcomes after limited with multiple staged revision surgeries are necessary.

## References

[1] Kurtz SM, Lau E, Watson H, Schmier JK, Parvizi J (2012). Economic burden of periprosthetic joint infection in the United States. J Arthroplasty..

[2] Kurtz S, Ong K, Lau E, Mowat F, Halpern M (2007). Projections of primary and revision hip and knee arthroplasty in the United States from 2005 to 2030. J Bone Joint Surg Am.

[3] Rupp M, Lau E, Kurtz SM, Alt V (2020). Projections of primary TKA and THA in Germany from 2016 through 2040. Clin Orthop Relat Res.

[4] Court-Brown CM, Duckworth AD, Clement ND, McQueen MM (2018). Fractures in older adults. A view of the future?. Injury.

[5] Klenerman L (2007). A history of osteomyelitis from the journal of bone and joint surgery: 1948 to 2006. J Bone Joint Surg Br.

[6] Orr HW (1927). The treatment of acute osteomyelitis by drainage and rest. J Bone Joint Surg Am.

[7] Frank RM, Cross MB, Della Valle CJ (2015). Periprosthetic joint infection: modern aspects of prevention, diagnosis, and treatment. J Knee Surg.

[8] Rajasekaran S (2007). Early versus delayed closure of open fractures. Injury.

[9] Brown PW, Kinman PB (1974). Gas gangrene in a metropolitan community. J Bone Joint Surg Am.

[10] Scharfenberger AV, Alabassi K, Smith S, Weber D, Dulai SK, Bergman JW, Beaupre LA (2017). Primary wound closure after open fracture: a prospective cohort study examining nonunion and deep infection. J Orthop Trauma.

[11] Hohmann E, Tetsworth K, Radziejowski M, Wiesniewski T (2007). Comparison of delayed and primary wound closure in the treatment of open tibial fractures. Arch Orthop Trauma Surg.

[12] Jenkinson RJ, Kiss A, Johnson S, Stephen DJ, Kreder HJ (2014). Delayed wound closure increases deep-infection rate associated with lower-grade open fractures: a propensity-matched cohort study. J Bone Joint Surg Am.

[13] Tiemann AH, Hofmann GO (2009). Principles of the therapy of bone infections in adult extremities. Strategies Trauma Limb Reconstr.

[14] Tiemann A, Hofmann G (2012). Wound irrigation within the surgical treatment of osteomyelitis. GMSInterdiscipPlastReconstrSurgDGPW.

[15] Metsemakers W, Morgenstern M, McNally M, Moriarty T, McFadyen I, Scarborough M, Athanasou N, Ochsner P, Kuehl R, Raschke M (2018). Fracture-related infection: a consensus on definition from an international expert group. Injury.

[16] Müller ME, Nazarian S, Koch P (1987). Classification AO des fractures: les os longs.

[17] Rupp M, Kern S, Weber T, Menges TD, Schnettler R, Heiß C, Alt V (2020). Polymicrobial infections and microbial patterns in infected nonunions–a descriptive analysis of 42 cases. BMC Infect Dis.

[18] Metsemakers W, Kuehl R, Moriarty T, Richards R, Verhofstad M, Borens O, Kates S, Morgenstern M (2018). Infection after fracture fixation: current surgical and microbiological concepts. Injury.

[19] Riesgo AM, Liporace FA (2018). Strategies for management of periprosthetic joint infection. Bull Hosp Jt Dis.

[20] Prasarn ML, Ouellette EA, Miller DR (2010). Infected nonunions of diaphyseal fractures of the forearm. Arch Orthop Trauma Surg.

[21] Wu C-C (2011). Single-stage surgical treatment of infected nonunion of the distal tibia. J Orthop Trauma.

[22] McNally M, Ferguson J, Lau A, Diefenbeck M, Scarborough M, Ramsden A, Atkins B (2016). Single-stage treatment of chronic osteomyelitis with a new absorbable, gentamicin-loaded, calcium sulphate/hydroxyapatite biocomposite: a prospective series of 100 cases. Bone Joint J.

[23] Wu H, Shen J, Yu X, Fu J, Yu S, Sun D, Xie Z (2017). Two stage management of Cierny-Mader type IV chronic osteomyelitis of the long bones. Injury.

[24] MacKenzie EJ, Castillo RC, Jones AS, Bosse MJ, Kellam JF, Pollak AN, Webb LX, Swiontkowski MF, Smith DG, Sanders RW (2007). Health-care costs associated with amputation or reconstruction of a limb-threatening injury. J Bone Joint Surg Am.

[25] Frisvoll C, Clarke-Jenssen J, Madsen J, Flugsrud G, Frihagen F, Andreassen G, Bere T (2019). Long-term outcomes after high-energy open tibial fractures: Is a salvaged limb superior to prosthesis in terms of physical function and quality of life?. Eur J Orthop Surg Traumatol.

[26] Dohmen PM, Konertz W (2007) A review of current strategies to reduce intraoperative bacterial contamination of surgical wounds. GMS Krankenhhyg Interdiszip 2(2):Doc38 PMC2831242 20204082

[27] Dąbrowska AM, Słotwiński R (2014). The immune response to surgery and infection. Cent Eur J Immunol.

